# Evidence for positive selection and recombination hotspots in *Deformed wing virus* (DWV)

**DOI:** 10.1038/srep41045

**Published:** 2017-01-25

**Authors:** A. Dalmon, C. Desbiez, M. Coulon, M. Thomasson, Y. Le Conte, C. Alaux, J. Vallon, B. Moury

**Affiliations:** 1 INRA, Unité Abeilles et Environnement, F-84000 Avignon, France; 2 UMT PRADE, F-84000 Avignon, France; 3 INRA, Unité Pathologie végétale, F-84000 Avignon, France; 4 ANSES, laboratoire de Sophia Antipolis, F-06902 Sophia Antipolis.

## Abstract

*Deformed wing virus* (DWV) is considered one of the most damaging pests in honey bees since the spread of its vector, *Varroa destructor*. In this study, we sequenced the whole genomes of two virus isolates and studied the evolutionary forces that act on DWV genomes. The isolate from a *Varroa*-tolerant bee colony was characterized by three recombination breakpoints between DWV and the closely related *Varroa destructor virus-1* (VDV-1), whereas the variant from the colony using conventional *Varroa* management was similar to the originally described DWV. From the complete sequence dataset, nine independent DWV-VDV-1 recombination breakpoints were detected, and recombination hotspots were found in the 5′ untranslated region (5′ UTR) and the conserved region encoding the helicase. Partial sequencing of the 5′ UTR and helicase-encoding region in 41 virus isolates suggested that most of the French isolates were recombinants. By applying different methods based on the ratio between non-synonymous (dN) and synonymous (dS) substitution rates, we identified four positions that showed evidence of positive selection. Three of these positions were in the putative leader protein (Lp), and one was in the polymerase. These findings raise the question of the putative role of the Lp in viral evolution.

Dramatic losses in honey bee (*Apis mellifera*) populations have been reported worldwide over the past 20 years^1^,^2^,^3^. Agricultural intensification (including the use of pesticides and a decrease in resources) and the spread of parasites, either alone or in combination, are often cited as potential drivers of honey bee colony decline or weakening^4^. Among parasites, the mite *Varroa destructor* is considered the most harmful pest in honey bee colonies^5^,^6^. This ectoparasite spread from Asia to the rest of the world by jumping from its original host, the Asian honey bee *Apis ceranae*, to the European honey bee *A. mellifera*^5^. Since this host shift, which was reported in the 1960 s, very few areas of the world are now free of this parasite^7^.

*Varroa* spread has contributed to a large increase in viral pathologies^8^,^9^. Among the twenty distinct virus species from the different genera that have been characterized in honey bees^10^, at least eight viral diseases have been associated with the presence of *Varroa destructor*^11^. Of these diseases, *deformed wing virus* (DWV, genus *Iflavirus*, family *Iflaviridae*, order *Picornavirales*) is often considered one of the most damaging viruses in honey bee colonies^12^,^13^,^14^,^15^,^16^. This virus most likely predicts bee winter mortality^17^. It induces symptoms including deformed and crippled wings and a dramatic decrease in bee longevity^18^. DWV is transmitted via the varroa mite^19^, and it is highly prevalent in countries that host *Varroa*^20^,^21^. Colony DWV load has been correlated with the level of *Varroa* infestation^15^. However, even in highly mite-infested colonies, bees do not systematically develop wing deformities, which suggests the presence of both overt and covert infections^18^. In fact, the mite acts as a(n) (i) virus multiplier^22^, (ii) vector by transmitting the virus directly into the bee hemolymph^19^, and (iii) “activator” by suppressing bee immunity and subsequently enhancing virus multiplication^23^,^24^,^25^. This pathosystem consequently consists of a tight triangular relationship between the pathogen DWV, the vector *V. destructor* and the host *A. mellifera*^26^.

Until recently, the virulence of DWV has been described primarily in terms of the global virus load of the infected honeybee colonies assessed using quantitative RT-PCR^27^. However, different genetic variants of DWV have been described^12^,^21^,^28^,^29^,^30^,^31^, and some studies have suggested that the evolution of the virus is driven by the vector^16^,^32^. The impact of virus recombination on virulence evolution is controversial. On the one hand, recent experiments have demonstrated the selection of a virulent variant in DWV populations after massive and repeated *Varroa*-mediated transmission^27^. Virulence was associated with a recombinant variant between DWV and the closely related *Varroa destructor virus-1* (VDV-1). On the other hand, Mordecai, *et al*.^29^ observed another DWV-VDV-1 recombinant variant in a *Varroa*-tolerant honey bee population and suggested that the emergence of recombinant variants may be the result of the adaptive evolution of DWV towards lower virulence to optimize its transmission by *Varroa* in the honey bee population.

The mechanisms associated with the emergence of wing deformities have not been completely described, and the respective roles of viral proteins in virulence are not known. In this study, we analyzed DWV and VDV-1 genome sequences to obtain clues regarding which viral proteins or genome regions are critical for virus adaptation. We first investigated whether recombination events might be involved in virus adaptation. Two complete sequences of DWV variants were obtained. We combined these sequences with additional sequences that were available in databanks; then we searched for recombination breakpoints and analyzed their distributions across the virus genome. Moreover, we compared both highly conserved and more variable genome regions among the partial virus sequences (42 isolates) that were obtained from colonies exposed to conventional *Varroa* management strategies and colonies tolerant to *Varroa*^33^. In addition, we searched for positively selected codons under the assumption that proteins that are critically involved in virus replication, transmission and adaptation to the host immune system could be evolving, at least in part, under positive selective pressure^34^,^35^,^36^.

## Results

### Complete sequences of two DWV/VDV-1 variants

We sequenced the complete genomes of two virus variants, one from a Varroa-infested colony with conventional management (DWV-Fr1) and a second from a colony that survived Varroa infestation for several years (RecVT-Fr1). Both colonies were located in Southern France (Table 1). These isolates resulted in sequences that were 10,143 and 10,104 nucleotides (nt) long, respectively, excluding their 3′ poly-adenylated tails. These sequences are available as GenBank accession numbers KX373899 and KX373900 (Table 1).

The two sequences were aligned with the 12 DWV/VDV-1 complete genomes, which were available in GenBank (Table 1). The alignment was 10,150 nucleotides long, which corresponded to the size of the VDV-1 genome (10,112 nucleotides^37^) plus gaps. A total of 2,129 variable nucleotide positions were identified in the whole dataset. Of these nucleotide positions, 1,669 were phylogenetically informative, and 460 corresponded to singletons. Among the segregating sites, the average pairwise nucleotide diversity between the sequences was π = 0.075, and the Tajima D test statistics rejected the neutrality hypothesis (D = 0.716). Variant DWV-Fr1 exhibited more than 95% similarity to the other DWV sequences, approximately 84% similarity to the VDV-1 sequence (Table 2) and 90 to 93% similarity to the recombinant variants described in Table 1. Variant RecVT-Fr1 showed the highest nucleotide identity with the DWV-VDV recombinants (95–96%) and lower identity with both the VDV-1 (90–92%) and the DWV (90–92%) sequences (Table 2).

**Figure 1:**
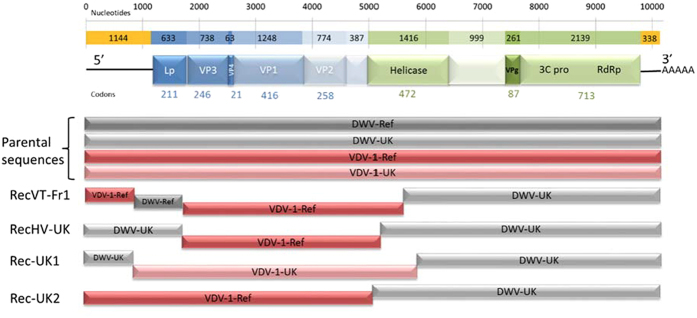
Scheme of the recombination events that were detected in 4 recombinant strains (RecVT-Fr1, RecHV-UK, Rec-UK1, and Rec-UK2). Total length of the genome is 10,140 nucleotides. The putative location of the proteins was determined according to de Miranda & Genersh (2010). First line: nucleotide scale; second line: number of nucleotides coding for each protein (regions coding for structural proteins are in blue, non-structural proteins are in green) or un-translated regions (yellow); third line: names of the corresponding proteins (same color legend as second line); fourth line: length of the proteins deduced from proteolysis sites (number of codons). Below: scheme of the recombinant genomes. The DWV sequences are shown in grey, and the VDV-1 sequences are shown in red (see Table 1 for the accession codes).

The open reading frame (ORF) of the variants DWV-Fr1 and RecVT-Fr1 started with the same “MAFS” amino acid motif also found in other DWV and VDV-1 sequences. The genome lengths differed from DWV-Ref because of deletions and insertions in the 5′ non-coding region. The putative proteins were positioned according to de Miranda and Genersch^18^: the first codon of the protein was inferred using proteolytic processing sites (Fig. 1).

### Diversity and phylogenetic analysis of full genome sequences

When comparing the number of nucleotide substitutions per site in each coding region, the helicase appeared to be the most highly conserved region in the genome, showing a minimum of 87.9% identity between DWV and VDV-1 (Table 3). The second most conserved region was the 3′ UTR (87.2%), which suggests that this non-coding region plays an important role in the virus. The most variable portion was the small putative structural leader protein (Lp, 211 codons), which had the highest divergence, showing 73.9% identity between DWV and VDV-1 (Table 3).

**Figure 2:**
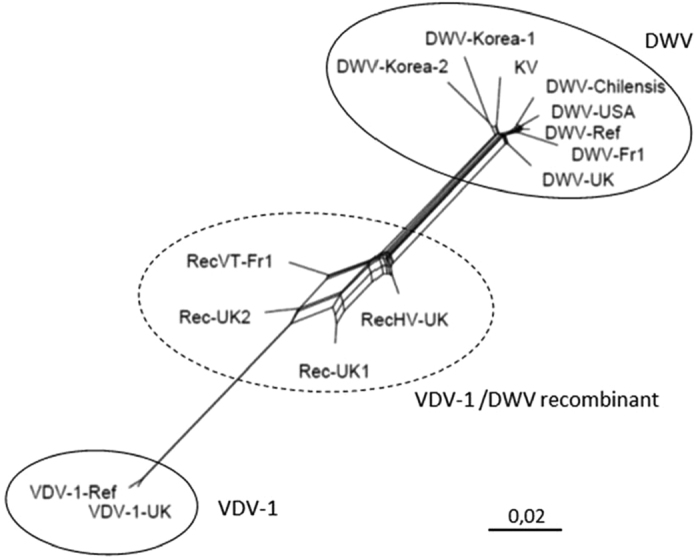
A split decomposition network of the complete genome sequences of DWV, VDV-1 and KV . This analysis involved 14 nucleotide sequences, 12 from the GenBank database and 2 (DWV-Fr1 and RecVT-Fr1) obtained during this study. The two distinct molecular groups (VDV-1 vs. DWV and KV) are indicated (solid lines) as are the acknowledged or putative VDV-DWV recombinants (dotted lines). The scale bar represents a genetic distance of 0.02.

The phylogenetic analysis showed that there were 2 well-supported clusters (Supplementary Fig. S2). One cluster included the original DWV (DWV-Ref), DWV-USA, DWV-Chilensis, variant DWV-Fr1, the Kakugo virus and the Korean variants. The other cluster grouped the VDV-1 isolates (VDV-1-Ref and VDV-1-UK). Between these clusters, 4 isolates presented an intermediate position with a low bootstrap support: three isolates acknowledged as DWV-VDV-1 recombinants (Rec-UK1, Rec-UK2 and RecHV-UK) and the French variant RecVT-Fr1 (Supplementary Fig. S2). A split decomposition analysis of the relationships between the complete sequences of all isolates confirmed that the 4 isolates Rec-UK1, Rec-UK2, RecHV-UK and RecVT-Fr1 had network-like rather than tree-like relationships with DWV and VDV, which is highly suggestive of recombination (Fig. 2). Variant RecVT-Fr1 was confirmed to be clearly distinct from the recombinant variant RecHV-UK^27^ and from the other recombinants previously described by Moore, *et al*.^28^ that prevailed in *Varroa destructor*-infested honeybee colonies.

### Recombination breakpoints

We examined recombination breakpoints in the entire dataset of complete sequences, without any assumption of putative parental sequences. A recombination breakpoint is the location in the genome where the RNA has been swapped from one parental sequence to another during RNA replication. Four of the variants analyzed in the study were DWV-VDV-1 interspecific recombinants (Table 4): variant RecVT-Fr1 (this study), the variant from the *Varroa*-infested colony RecHV-UK, variant Rec-UK1 and variant Rec-UK2. Three breakpoints were detected in the genome of RecVT-Fr1. Wherever a putative recombination breakpoint was observed, we sequenced the fragment encompassing this breakpoint to make sure it was not an artefact resulting from mixed infections. Thus, this isolate appeared to be a triple recombinant, with two VDV-1-related regions composed most of the 5′ UTR and the 5′ half of the ORF, and two DWV-related regions (Fig. 1).

In the whole dataset, eight highly significant recombination breakpoints were identified in the genomes of the four variants cited above (*p*-values < 10^−79^, Table 4). Two were found in the 5′ untranslated region, and six were found in the open reading frame (ORF; Fig. 1). All of these breakpoints were confirmed to be highly significant by at least six of the 7 different methods that were used. For each recombinant (RecHV-UK, Rec-UK1, Rec-UK2 and RecVT-Fr1), a breakpoint was identified in the region encoding the helicase (Table 4), but they never resulted in an aminoacid change (data not shown). The recombination breakpoints in this region were located between positions 5710 and 5716 (RecVT-Fr1) and 5800 and 5836 (Rec-UK1) and were clearly different. In contrast, because this region is highly conserved, we could not determine with high precision the position of the recombination breakpoints in variants RecHV-UK [5089–5134] and Rec-UK2 [5134–5167], and we could not exclude that the recombination breakpoint in these two variants was inherited from the same ancestral recombination event. Consequently, we did not consider these two recombination breakpoints to be independent. The breakpoints in the Leader protein (Lp) coding sequences were very close (positions [1753–1759] in variant RecVT-Fr1 and [1685–1688] in variant RecHV-UK), but the recombination intervals that were estimated using RDP or after a visual examination did not overlap. On the contrary, the breakpoints identified in the 5′ UTR of variants RecVT-Fr1 (positions [930–933]) and Rec-UK1 (positions [933–948]) could be located at the same position (Table 4, Fig. 1). However, we determined that they corresponded to two independent recombination events because the parental sequences located on each side of the breakpoints corresponded to opposite virus species, DWV or VDV-1, in the two variants (Fig. 1). In all, because two of the 8 breakpoints initially detected corresponded to a unique event, seven independent recombination breakpoints were mapped in the complete genome dataset.

**Figure 3:**
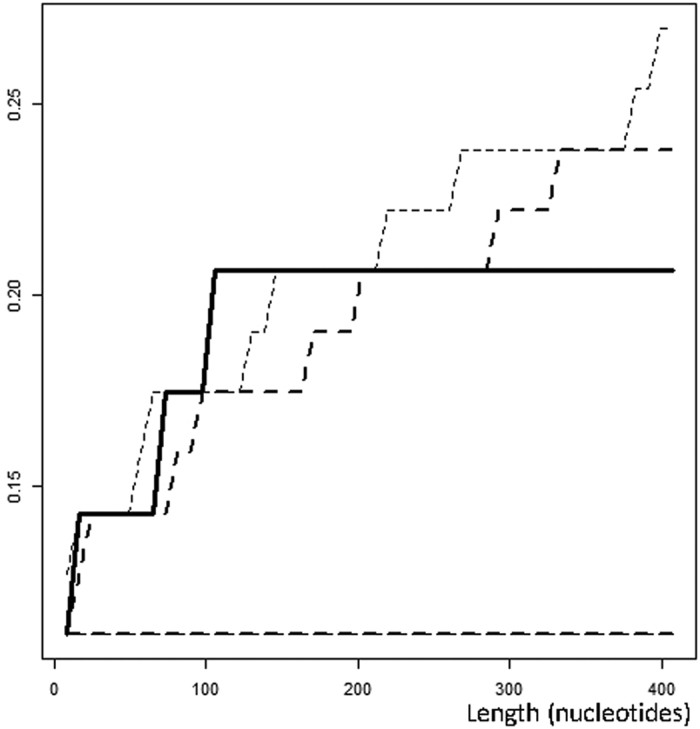
Distance probability between breakpoints in a 100 and 200 nucleotides range. Long dot lines indicate the 5% significance threshold, and short dot lines indicate the 2.5% threshold. Continuous line results are shown from our dataset.

Next, we examined the distribution of these breakpoints across the genome (Fig. 1, Table 4), and tested the pattern of recombination events to determine whether complete spatial randomness (CSR) was present. A significantly (*p-*value < 0.025) higher number of recombination breakpoints was detected at distances of 100 to 150 nucleotides between breakpoints (Fig. 3) than for the distribution of distances between breakpoints expected by chance. A similar excess was observed at distances < 150 nucleotides from the nearest neighboring breakpoint, which emphasizes the importance of small distances (Supplementary Fig. S2a). These excesses in the observed distributions compared with the calculated random distributions reveal the presence of significant recombination hotspots. Randomness was also rejected at the 2.5% significance level with regard to the distances between any site of the genome and the nearest breakpoint for distances of >100 nucleotides. This finding emphasizes the importance of empty space^38^ and reveals the presence of recombination coldspots (Supplementary Fig. S2b).

### Partial sequences obtained from isolates with different origins and recombinant prevalence

Partial sequences from the 5′ UTRs were obtained from 23 isolates collected from 6 locations in France (Supplementary Table S1). Three isolates (F13CO05, F14PA010 and F14PR108) were excluded from the analysis because they showed a mixture of sequences (data not shown). In this 5′UTR region, two variants, RecVT-FR2 and RecVT-FR3, showed evidence of one significant recombination breakpoint (*p*-value < 10^−3^ using at least three of seven methods; Table 4). Interestingly, variant RecVT-FR2 was closely related to VDV-1 at the 5′ side of the recombination positions [358–379] and to DWV at the 3′ side, whereas variant RecVT-FR3 was close to DWV at the 5′ side of the recombination positions [270–280] and to VDV-1 at the 3′ side. Thus, in addition to the 7 DWV-VDV recombination breakpoints analyzed above, we identified two new independent recombination breakpoints in the 5′UTR.

In all, of the 22-French isolates analyzed (including DWV-Fr1 and RecVT-Fr1), we obtained 18 sequences of 5′ UTRs (17 plus variant RecVT-Fr1) that were closely related to VDV-1 and 4 sequences (RecVT-Fr2, DWV-Fr3, DWV-Fr2, and DWV-Fr1) that were closely related to DWV. All of the partial sequences obtained in the helicase region were characterized as DWV. When isolates were closely related to DWV in the helicase region and closely related to VDV-1 in the 5′ UTR, we inferred that a recombination event had occurred somewhere between the two genome regions. Three of the 18 VDV-1-like 5′ UTR sequence isolates could not be amplified in the helicase region. Two were discarded from the recombinant prevalence calculations, but one of these isolates (RecVT-Fr3) was a recombinant in the 5′ UTR region (Table 4). Thus the latter was still considered a recombinant variant (Supplementary Table S1). Moreover, given that RecVT-Fr2 that was closely related to DWV in the 5′ UTR was a recombinant (Table 4), we identified 17 such recombinants of the 20 French isolates (Supplementary Table S1), indicating a 85% recombinant prevalence in our sample. These data suggest that there is recombinant predominance in France. Neither the Italian, the Argentinian nor the Canadian variants could be amplified in the 5′ UTR (Supplementary Table S1), and we were therefore unable to determine whether they were recombinant or not. The lack of amplification of the 5′UTR of these isolates might be because the primers showed higher affinity for VDV-1 or recombinant isolates. However, the 5′ UTRs of the three French DWV variants (DWV-Fr1, DWV-Fr2andDWV-Fr3) were amplified and sequenced, and they did not show any evidence of recombination.

### Detection of positive selection in the leader protein and the polymerase

**Figure 4:**
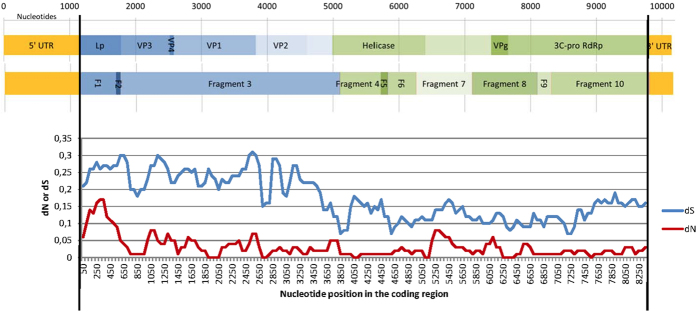
Scheme of the fragments (F1 to F10) that were analyzed to estimate the rates of synonymous (dS, in blue) and non-synonymous (dN, in red) substitutions. Substitution rates along the DWV ORF and the dN and dS values were estimated using SNAP (http://www.hiv.lanl.gov)^61^. The *x*-axis represents the nucleotide positions in the ORF, and the *y*-axis represents the average rate of synonymous or non-synonymous substitution rates, as estimated using a 50-codon sliding window.

The complete polyprotein coding sequence was split into regions devoid of recombination breakpoints (Fig. 4) and examined for positive selection. Along the whole ORF (2873 codons), we calculated estimates of the rates of synonymous substitutions per site (dS) and non-synonymous substitutions per site (dN) for each codon. Figure 4 shows the positions of these fragments in the ORF. Fragments 1 to 3 corresponded to structural proteins and the 5′ end of the helicase-coding region, and fragments 4 to 10 encompassed non-structural proteins. A sliding window analysis revealed that the region encoding the putative leader protein (Lp) exhibited a higher nonsynonymous substitution rate than the other proteins (Fig. 4).

Four potentially positively selected codon positions were identified along the polyprotein by at least two methods (Table 5). Three of these positions were located in the Lp (codon sites 21, 57 and 107), which is consistent with the higher rate of nonsynonymous substitutions observed in the first 150 to 400 nucleotides of the ORF (0.12 ≤ dN ≤ 0.17, Fig. 4). In the 3′ part of the ORF (fragment 10, that corresponded to the 3′ end of the putative polymerase-coding sequence), both datasets (one of 17 sequences and the other of 48 sequences corresponding to a shorter region) identified codon position 2,838 to be under positive selection. Only one additional codon site or particular branch in the phylogenetic tree was found to be under positive selection using the MEME and Branch-REL methods (codon position 1627 in fragment 6). It was also identified using the IFEL method but not confirmed via the FEL, SLAC or FUBAR methods.

## Discussion

We obtained the complete sequences for two DWV isolates from the same site in France. One of them, variant RecVT-Fr1, originating from a *Varroa*-tolerant colony, was a complex recombinant between DWV and VDV-1 that included three recombination breakpoints. This is the first description of a triple recombinant because all previously described recombinants comprised only one or two recombination breakpoints^27^,^28^,^29^,^31^.

DWV and VDV-1 were initially considered different species based on the 84% nucleotide identity of their genomes and their isolation from different hosts^37^,^39^. However, both virus species were later shown to infect both *Varroa* and bees^31^. Moreover, the species cannot be distinguished using specific biological properties. Identity thresholds have been set as species demarcation criteria by the International Committee on Taxonomy of Viruses (ICTV) based on the distribution of pairwise sequence comparisons. Regarding the pairs of sequences obtained from the same species, when two peaks were identified, the first likely corresponds to a “strain” comparison, whereas the second likely corresponds to a “variant” comparison^40^. If we apply this rule to DWV and VDV-1, then we can consider the first peak of identity from 95.8% to 99.2% to be characteristic of the variants (Table 1), whereas identity levels from 84.1% to 84.9% are characteristic of the DWV and VDV-1 “strains”. This proposal corroborates the conclusions of Mordecai, *et al*.^29^, who proposed that we name the original DWV “strain” (DWV-Ref) “type A” and the VDV-1 “strain” “type B”. A type C variant was recently characterized in bee populations tolerant to *Varroa* in Swindon (UK)^41^. These variants formed a distinct and separate branch in the DWV phylogeny. Consequently, types A, B and C could be considered three different strains within a single virus species that is composed of both DWV and VDV-1. Several recombinants issued from the A, B and C types have already been described^27^,^28^,^29^,^31^ (and recently McMahon, *et al*.^42^). Based on our French samples, we estimate that the minimal prevalence of recombinants is 85%. The widespread occurrence of these recombinants and the diversity of the observed recombination breakpoints are arguments in favor of considering DWV and VDV-1 to be different variants that belong to a single species. Moreover, because recombination was frequent, we cannot exclude the possibility that other recombination breakpoints would be revealed in a larger dataset. The taxonomy may therefore need to be revised to take into account the proximity of the previously described DWV and VDV-1 “species” and to consider DWV as a single species that includes different strains or types.

The nine recombination breakpoints that were identified in the virus genomes analyzed in this study (Table 4) demonstrate two things. First, recombination can occur at different sites in the 5′ UTR, the 3′ end of Lp and the 5′ end of the helicase-coding region (Fig. 1). Second, recombination breakpoints are not randomly distributed but are instead significantly aggregated (Fig. 3), indicating the presence of two hotspots. One hotspot is located in the helicase-coding region, and the other one is located in the Lp coding region and 5′ UTR. Hence, these hotspots allow us to define roughly three genomic clusters: the 5′ UTR, the region encoding structural proteins (Lp and capsid proteins) and the region encoding non-structural proteins. These hotspots are therefore in agreement with the “three module” functional nature of the *Picornaviridae* genome, favoring recombination at the junctions of these functional domains (or next to their boundaries) and not within the domains themselves^43^.

Recombination hotspots might be explained by the recombination event itself, which might not occur with the same probability along the virus genome, and/or by the fitness of the recombinants, that might differ depending on the location of the breakpoints.

A high degree of similarity in the parental sequences can promote recombination events^44^. Interestingly, the identity between virus variants was highest in the helicase-coding region (minimum 87.9%, Table 3), where recombination was shown to have occurred in all of the completely sequenced recombinants. However, this hypothesis, which proposes genetic proximity, does not hold for recombination breakpoints that are localized in the 5′ UTR or the Lp-coding region, in which the degree of similarity was low (Table 3).

A second hypothesis proposes that the secondary structures of the viral RNA may favor recombination, as has been shown in the human poliovirus^45^ and bee dicistrovirus^46^.

A third hypothesis is not linked to the probability of recombination occurring along the genome and is instead linked to the fitness of the generated recombinants. The advantage of recombination has largely been debated. Usually, recombinants are thought to be less fit than their parents, on average, and this has been demonstrated in several RNA viruses^47^,^48^. The fact that we observed recombination hotspots close to the frontiers of the three proposed modules of the DWV genome suggests that these hotspots may result from selective pressure favoring these recombinants because they globally preserve the three functional modules that are essential for virus survival and dispersal^49^. In addition, virus diversity might be influenced by a host RNA interfering (RNAi) response during infection. Some recombinants might be more susceptible than others to the RNAi anti-viral defense of the bees because they exhibit sequences that are targeted by virus-derived small interfering RNAs (vsiRNAs). Such a reduction in virus load was shown for DWV when feeding double-stranded viral RNA to induce the production of vsiRNAs^50^. Recombinants might escape the RNAi targeting parental sequences if recombination breakpoints occur in the targeted sequences, thereby enhancing fitness.

Interestingly, the RecVT-Fr1 recombinant originated from a colony that survived a *Varroa* infestation for over 17 years without any *Varroa* management^33^. Direct sequencing of PCR products from 30 bees did not reveal mixtures of sequences, and these results indicated a predominance of this recombinant in the colony. In addition, we identified other recombinant variants (Fig. 1) in other *Varroa*-tolerant honey bee populations that originated from completely distinct geographic origins (RecVT-Fr2, RecVT-Fr3, RecVT-Fr4) and possibly from different honey bee populations, suggesting that DWV recombination may be at least partly linked to *Varroa* tolerance and/or virus virulence. For such a link to occur, there should be a causal relationship between recombination, virulence and virus fitness. It is widely thought that virulence is maintained in parasite populations as a consequence of parasite multiplication in the host, which, in turn, is required for efficient transmission. In that sense, virulence is advantageous for the parasite, at least in the short term. However, if virulence is too high, the parasite may kill its host too rapidly to ensure efficient transmission. Consequently, over the longer term, including a long chain of transmission events, lower virulence may be selected. The trade-off model^51^ proposed that virulence reaches an equilibrium between the somewhat contradictory requirements of parasite within-host accumulation and between-host transmission. Recombination, by decreasing the accumulation of the virus within its bee host, may accelerate a decrease in virus virulence and, over the long term, thereby favor its transmission. In this model, non-recombinant viruses with high virulence may be favored over the short term after virus introduction whereas recombinant variants with lower virulence may be selected for over the long term.

However, no clear link has been established in the literature between DWV/VDV-1 recombination and virus virulence, accumulation or transmission. By exposing larvae, either orally or via *Varroa*, to a mixture of DWV variants, Ryabov, *et al*.^27^ identified a highly virulent recombinant between the original DWV and VDV-1 types that resulted in deformed wing symptoms in adult bees. Accordingly, a study of Devon (UK) colonies suffering overwintering colony losses resulted in the identification of a type C recombinant between the original DWV and DWV that was admixed to other types of variants^41^. In our French samples, a majority represented recombinants, but there was no obvious link between the observed virulence of the virus and the *Varroa* management strategy. Clearly, further studies are required to test this hypothesis. For example, the prevalence of recombinants and their virulence compared to the parental viruses could be tracked over years as a function of *Varroa* management strategies to determine whether they affect the efficiency of virus transmission.

Superinfection exclusion (SIE) may also have contributed to the emergence of DWV/VDV-1 recombinants^29^. SIE is a phenomenon by which a primary viral infection prevents a secondary infection by the same or a genetically closely related form of the virus^52^. SIE was recently described in a *Varroa*-tolerant honeybee population that was isolated in Swindon (UK). Although it was present in the vector, the highly virulent variant could not be maintained in the colonies, and the less virulent variants remained the most widely prevalent. By favoring the maintenance of virus populations with a lower virulence and/or a lower fitness from other virus competitors with higher virulence/fitness, SIE may have accelerated the emergence of recombinants.

Our analysis shows evidence of recombination and positive selection in the Lp-coding region. The proximity of most of the positively selected codon positions to one of the two recombination hotspots in, or close to, the Lp-coding region strengthens the functional importance of the Lp for DWV-VDV-1 adaptation. However, it should be emphasized that a higher number of sequences were analyzed in the Lp-coding region, which provided a higher statistical power for detecting positive selection in this region than in the other regions of the genome. As described for other picornaviruses, the Lp protein has been implicated the cleavage of the translation initiation factor eIF4G. This event inhibits cap-dependent translation in infected host cells and stimulates viral IRES (internal ribosome entry site) activity^53^. Because they show evidence of positive selection, some amino acids at positions 21, 57 and 107 of the Lp region may be involved in inhibiting cap-dependent mRNA translation in the host, possibly stimulating IRES activity and thereby favoring virus replication in both the bee host and the *Varroa* vector. The presence of recombination breakpoints between the 5′ UTR and the Lp-coding region indicates that the 5′ UTR may also be involved in virulence. The highly virulent isolate RecHV-UK has a 5′ UTR similar to that of DWV, and this may increase the efficiency of the translation of the viral polyprotein.

In addition, all known recombinants contain a region that encodes non-structural proteins (NS) similar to DWV. The recombinants were shown to be more efficient at replicating than the parental variants^23^,^29^. The DWV replication complex may be essential for a high rate of replication because it is more adapted to its original bee host. The evidence for positive selection at the 3′ end of the putative polymerase-encoding sequence (the codon at position 2838) may confirm this selective advantage.

By combining phylogenetic and molecular evolutionary analyses, in this study we identified 9 independent recombination breakpoints and showed that they were not randomly distributed across the DWV genome. In addition, several codon sites were shown to be under positive selective pressure in the region encoding structural proteins (Lp) and non-structural proteins (polymerase). In human diseases, it has been suggested that vector-transmitted pathogens would indirectly increase virulence by increasing within-host genetic variation^54^. Here, we provide evidence for the presence of genetic diversity in virus populations, and we show that this diversity was generated by recombination events that globally preserved the modular structure of the genome. These events might have helped the virus adapt to both its honey bee host and its *Varroa* vector.

## Material and Methods

### Bee sampling

In this study, we use the word “isolate” to indicate the colony origin and “variant” to refer to genetic characteristics. Bees were collected during the summer of 2013 from one colony with deformed wing symptoms (isolate DWV-Fr1) located in the experimental apiary of INRA Avignon (South-Eastern France). Another isolate that originated from a *Varroa*-tolerant colony^33^ was collected from the same location in September 2013 (isolate RecVT-Fr1). A total of 32 samples were also collected from 6 areas in France, and 11 were collected from Italy, Argentina and Canada (Supplementary Table S1). The bees were maintained alive on ice, rapidly frozen and stored at −20° or −80 °C, except for the samples obtained from Argentina, which were lyophilized, or Canada, which were stored in 98° ethanol.

### RNA extraction

Bees from the same colony were pooled and then ground according to different protocols depending on the number of bees per sample. Pools ≤ 10 bees were homogenized in 800 μl of QIAzol (Qiagen) containing one 0.8 cm-diam. bead in a 2 ml tube and a TissueLyser (Qiagen) (four times for 30 seconds each at 30 Hz at 30-second intervals). The tube was then centrifuged for 2 min at 12,000 g at 4 °C, and the supernatant was collected into a new tube to be processed for RNA extraction. Pools >10 bees were placed in Bioreba plastic bags containing PBS at 4 °C (1 ml/10 bees), and the bees were then homogenized using a hand model (Bioreba) that was fixed on a drilling machine. A 500-μl volume of the extract was immediately added to 500 μl of QIAzol (Qiagen) and vortexed, and the solution was then processed for RNA extraction. RNA was extracted using a RNeasy Plus Universal mini kit (Qiagen, Courtaboeuf, France) according to the manufacturer’s instructions. The final suspension volume was 100 μl. RNA was quantified using a Nanodrop^®^ spectrophotometer.

### RT-PCR and sequencing

Reverse transcription was performed using 1 μg RNA in 20 μl reaction volumes containing random primers according to the manufacturer’s protocol (High capacity RNA to cDNA, Life technologies). Three microliters of ten-fold diluted cDNA was mixed with 1.5 mM MgCl_2_, 10 pmol of each primer, 0.2 mM dNTP, 1X green Taq buffer and 1 unit of GoTaq G2 Flexi DNA polymerase (Promega) in a 25 μl total volume. PCR reactions were performed using an Eppendorf or MJ Research thermocycler. The following program was used: one cycle at 94 °C for 2 min, 35 cycles at 94 °C for 30 s, 55 °C for 30 s, and 72 °C for 45 s, and a final elongation cycle at 72 °C for 10 minutes. The reactions were then held at 10 °C. For fragments longer than 700 bp or 1000 bp, the elongation step was lengthened to 1 or 2 min, respectively. The primers used to perform the sequencing reactions are listed in Supplementary Table S2. The primers used to partially sequence the helicase-encoding region were DWV-5992f and DWV-6693r^21^. The primers used to sequence the 5′ UTR were DWV-UTR1f (5′-CGATTTATGCCTT(C/G)CATAGCG-3′) and DWV-1795r (5′-TACGTTCTTGCTCCAGCGCC-3′). The annealing temperature was raised to 62 °C for these reactions.

Five microliters of each PCR product was run on a 1% agarose gel to determine purity and band size. The remaining PCR products were then sent to Genoscreen (Lille, France) for purification and direct sequencing. The sequences were used to define specific primers for subsequent sequencing. The sequences of the 5′ and 3′ extremities of isolate DWV-Fr1 were obtained using 5′ RACE according to the manufacturer’s instructions (Invitrogen, Paisley, UK) and an oligo (dT) primer, respectively.

### Sequence analysis

Sequences were assembled using CAP3^55^ with manual corrections when needed. The sequences were aligned using the GenBank sequences for DWV and VDV-1 (DWV: NC_004830 [DWV-Ref], AY292384 [DWV-USA], JQ413340 [DWV-chilensis], GU109335 [DWV-Warwick], JX87830 [DWV-Korea 1], and JX878304 [DWV-Korea-2]; KV: AB070969 [Kakugo virus]; and VDV-1: AY251269 and NC_006494; Table 1). Recombination was analyzed using RDP4 (including RDP, Geneconv, BootScan, MaxChi, Chimaera, SiScan, and 3-Seq) and GARD^56^ software. All genomes appearing to be putative recombinants of DWV and VDV-1 (including RecVT-Fr1 [this study], RecHV-UK, Rec-UK1, and Rec-UK2, see below) were tested separately using potential parental sequences in RDP4 using the seven algorithms listed above.

We tested the pattern of recombination events along the virus genome to identify whether there was complete spatial randomness (CSR) in the set of seven independent recombination breakpoints that were identified from the alignment of ten complete genome sequences, as described for spatial point patterns^38^,^57^. Three parameters were tested to determine CSR: (1) the pairwise distance between recombination breakpoints, (2) the distance to the nearest neighboring breakpoint, which emphasizes the importance of small distances, and (3) the distance of any site of the genome to the nearest breakpoint, which emphasizes the importance of empty space^38^. The estimations of these three parameters for the identified recombination breakpoints were compared to 1000 simulations that were performed under complete randomness using R software (http://cran.r-project.org/).

The phylogenetic and sequence diversity analyses were performed using MEGA version 6 58. The best substitution model was selected using the Hyphy package (http://www.datamonkey.org). A neighbor-joining tree was built using the 14 complete nucleotide sequences available, with boostrap resampling (1,000 iterations). Because recombinants were suspected, a split decomposition analysis was performed using SplitsTree4.7 59 to obtain a split network representing ambiguous signals in the dataset. The Tajima D test was performed and the average pairwise nucleotide diversity (π) was calculated using MEGA version 6. To investigate whether the DWV populations had been subject to positive or negative selection, we calculated the ratios of non-synonymous (dN) and synonymous (dS) substitution rates on the alignments of the coding genome regions. Positive selection was assumed when dN/dS > 1, and negative selection was inferred when dN/dS < 1. Ambiguous codon positions were discarded from the analysis. For each genome fragment, we checked for partial DWV sequences in GenBank to enlarge the dataset. The analysis for positive selection was performed on the Web server using the HyPhy Package with the FEL (fixed-effect likelihood), IFEL (Internal fixed-effect likelihood), REL (random effect likelihood), FUBAR (Fast Unconstrained Bayesian AppRoximation), SLAC (single-likelihood ancestor counting), MEME (mixed effect model evolution) and Branch-REL methods, incorporating the best nucleotide substitution model, as defined by the HyPhy Package. MEME assumes that some branches may be under positive selection while others are under negative selection, allowing dN/dS to vary along branches according to a 2-bin distribution. MEME assumes a random effect of the lineages (branches of the phylogenetic tree) on the resulting codon sites that were under positive selection. Branch-REL looks for lineages in which a proportion of codon sites evolved with a dN/dS > 1 without making any assumptions about lineage identification or what occurred in the rest of the lineages^60^. All other methods assume that dN/dS varies across codon sites but not across lineages. All positive selection analyses were performed separately for each genome segment devoid of recombination breakpoints.

## Additional Information

Accession codes: KX373899, KX373900 (complete sequences). KX373901 to KX373936 (sequences in the helicase coding region). KX373937 to KX373956 (5′ UTR sequences).

**How to cite this article**: Dalmon, A. *et al*. Evidence for positive selection and recombination hotspots in *Deformed wing virus* (DWV). *Sci. Rep.* **7**, 41045; doi: 10.1038/srep41045 (2017).

**Publisher's note:** Springer Nature remains neutral with regard to jurisdictional claims in published maps and institutional affiliations.

## Supplementary Material

Supplementary Material

## Figures and Tables

**Table 1:** Description of the full genome sequences in Genbank.

Name in this study	Accession number in Genbank	Name in the original study	Geographic origin	Length (nucleotides)	Recombinant (R) or parental sequence (P)	Associated symptoms (if described)	Originating from a *Varroa*-tolerant colony	Authors, year published in Genbank
DWV-Fr1	KX373899	85	France	10,143	P		No	Current study
RecVT-Fr1	KX373900	123	France	10,104	R		Yes	Current study
RecHV-UK	KJ437447	DWV isolate Varroa-infested-colony-DJE202	United Kingdom	10,167	R	Highly virulent	No	Ryabov *et al*.^27^
DWV-Chilensis	JQ413340	DWV isolate Chilensis A1	Chili	10,171	P		No	Barriga *et al*. 2012
DWV-Ref	NC_004830	DWV	Italy	10,140	P	Deformed wings	No	Lanzi *et al*.^12^
Rec-UK2	HM067438	VDV-1-DWV-no-9	United Kingdom	10,154	R		No	Moore *et al*.^28^
Rec-UK1	HM067437	DWV isolate VDV-1-DWV-No-5	United Kingdom	10,149	R		No	Moore *et al*.^28^
DWV-USA	AY292384	DWV isolate PA (Pennsylvania)	USA	10,166	P	Deformed wings	No	Lanzi *et al*.^12^; de Miranda, J. *et al*. 2003
DWV-Korea-2	JX878305	DWV strain Korea-2	Korea	10,114	P		No	Reddy *et al*. 2013
DWV-Korea-1	JX878304	DWV strain Korea-1	Korea	10,111	P		No	Reddy *et al*. 2013
DWV-UK	GU109335	DWV isolate Warwick-2009	United Kingdom	10,167	P		No	Bouleau Jamois *et al*. 2009
KV	NC_005876	KV	Japan	10,152	P	Aggressive workers	No	Fujiyuki, T. 2001
VDV-1-Ref	NC_006494	VDV-1	Netherlands	10,112	P		No	Ongus *et al*.^37^
VDV-1-UK	KC786222	VDV-1_Ox	United Kingdom	10,089	P		No	Wang *et al*.^62^

“Name in this study” uses “Rec” for DWV/VDV-1 recombinant isolates and includes references to geographic origin. “VT” was applied when the isolate was collected from a *Varroa*-tolerant colony, and “HV” indicates a highly virulent strain.

**Table 2:** Estimates of nucleotide identity between sequences (% of the number of base substitutions per site between sequences).

	DWV-Fr1	RecVT-Fr1	RecHV-UK	DWV-CHILENSIS	DWV-Ref	Rec-UK2	Rec-UK1	DWV-USA	DWV-KOREA-2	DWV-KOREA-1	DWV-UK	KV	VDV-1-Ref	
DWV-Fr1														
RecVT-Fr1	91.5%													
RecHV-UK	93.0%	95.9%												
DWV-CHILENSIS	97.6%	91.7%	93.0%											
DWV-Ref	98.6%	92.3%	93.8%	98.6%										
Rec-UK2	90.2%	96.1%	95.2%	90.4%	91.1%									
Rec-UK1	90.8%	95.0%	96.9%	90.9%	91.5%	96.5%								
DWV-USA	98.1%	92.0%	93.3%	98.5%	99.2%	90.6%	91.1%							
DWV-KOREA-2	96.3%	90.6%	92.3%	96.5%	97.2%	90.0%	90.7%	96.9%						
DWV-KOREA-1	95.8%	90.2%	92.0%	96.1%	96.7%	89.7%	90.5%	96.5%	96.6%					
DWV-UK	97.3%	91.2%	93.7%	97.4%	98.3%	90.7%	91.8%	97.8%	96.7%	96.4%				
KV	96.6%	90.8%	92.7%	96.9%	97.6%	90.4%	91.1%	97.4%	96.9%	96.5%	97.4%			
VDV-1-Ref	84.4%	91.3%	89.5%	84.6%	84.9%	92.4%	91.6%	84.6%	84.2%	84.1%	84.6%	84.5%		
VDV-1-UK	84.6%	91.7%	89.8%	84.8%	85.1%	92.5%	91.8%	84.8%	84.5%	84.3%	84.9%	84.8%	99.4%	

Analyses were conducted using the p-distance method. The analysis involved 14 nucleotide sequences. All ambiguous positions were removed for each sequence pair. There were 10,150 positions in the final dataset. Evolutionary relationships were analyzed in MEGA6^58^.

**Table 3:** Estimates of nucleotide identity between DWV, VDV-1 and recombinant sequences in different genomic regions.

Complete sequences	Max	Min	Mean
	99.4%	84.1%	92.4%
5′UTR	99.5%	82.9%	91.9%
LP	99.4%	73.9%	86.5%
VP3	99.3%	83.3%	90.7%
VP4	99.6%	86.3%	94.6%
VP1	99.8%	84.1%	91%
VP2	100.0%	84.1%	91.3%
Helicase	99.5%	87.9%	94.7%
VPg	99.6%	86.3%	94.6%
3C-pro RdRp	99.3%	85.3%	94.0%
3′UTR	100.0%	87.2%	95.0%

Minimum distances correspond to comparisons between DWV and VDV-1 sequences and the percentages for 14 full genome sequences. The differences per site between sequences analyzed using the p-distance method are shown. All ambiguous positions were removed for each sequence pair. A total of 10,150 positions in the final dataset.

**Table 4:** Recombination breakpoints.

Isolate	Major parent	Minor parent	Beginning (or unique) breakpoint	Ending breakpoint	*P*-value
RecVT-Fr1	VDV-1-Ref	DWV-Ref	[930–933]	[1753–1759]	1.07 × 10^−80^
RecVT-Fr1	DWV-UK	VDV-1-Ref	[1753–1759]	[5710–5716]	1.27 × 10^−160^
RecHV-UK	DWV-UK	VDV-1-Ref	[1685–1688]	[5089–5134]	1.27 × 10^−160^
Rec-UK1	DWV-UK	VDV-1-UK	[933–948]	[5800–5836]	2.51 × 10^−168^
Rec-UK2	DWV-UK	VDV-1-Ref	[5134–5167]		1.27 × 10^−160^
RecVT-Fr2 F14SA066	DWV-UK	VDV-1-Ref	[358–379]		8.85 × 10^−7^
RecVT-Fr3 F15SA228	DWV-USA	RecVT-Fr2	[270–280]		9.21 × 10^−8^

Positions across the genome are indicated in brackets (NC_004830; DWV-Ref in this study is shown as a Reference). *P*-values were calculated using RDP. The potential parent sequences were DWV-Fr1, RecVT-Fr1, DWV-USA, DWV-Ref, DWV-chilensis, DWV-UK, DWV-Korea 1, DWV- DWV-Korea-2, RecHV-UK, Rec-UK1 and Rec-UK2 (DWV-VDV-1 recombinants) as well as VDV-1-Ref, VDV-1-UK and KV (Kakugo virus).

**Table 5:**
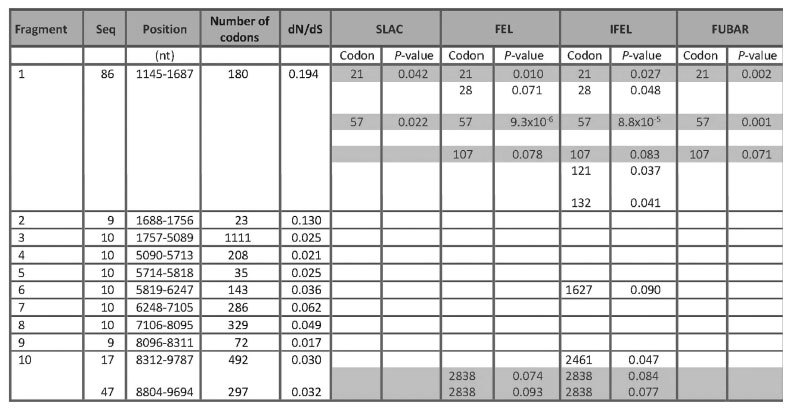
Codon positions in the ORF of DWV, VDV-1 or recombinants that were affected by positive selection according to the four methods implemented in the HyPhy software.
