# Supplementary material for: Evidence for positive selection and recombination hotspots in Deformed wing virus (DWV)
Source: Sci Rep. 2017 Jan 25;7:41045. doi: 10.1038/srep41045 (PMC5264398; doi:10.1038/srep41045)

Evidence for positive selection and recombination hotspots in *Deformed wing virus* (DWV)

A. Dalmon, C. Desbiez, M. Coulon, M. Thomasson, Y. Le Conte, C. Alaux, J. Vallon, and B. Moury

Supplementary Table S1 - Origin of the sequenced virus isolates and GenBank accession numbers. "-" indicates that no PCR product was obtained, "+" indicates that a PCR product was obtained but could not be sequenced. NT, not tested. Alsace, Corsica, Levant, PACA, Sarthe, and Paris are regions, departments or localities in France.

**Variant name**	Recombinant (R) or parental sequence (P)	**Laboratory code**	Origin year	Pools of n bees n=	UTR sequence	Helicase sequence
DWV-Fr1	P	85	PACA 2013	10	KX373899	KX373899
RecVT-Fr1	R	123	PACA 2013	10	KX373900	KX373900
	R	F13AL022	Alsace 2013	6	KX373939	KX373906
	R	F13AL023	Alsace 2013	6	KX373946	KX373907
	Unknown	F13CO05	Corsica 2013	10	+	KX373915
	R	F13CO06	Corsica 2013	10	KX373945	KX373914
DWV-Fr2	P	F13CO08	Corsica 2013	10	KX373955	+
	Unknown	F13LE024	Levant 2013	6	KX373948	-
	Unknown	F13LE026	Levant 2013	6	-	KX373916
	Unknown	F13PA010	PACA 2013	10	+	KX373919
	Unknown	F13PA028	PACA 2013	7	-	KX373920
	R	F14PA038	PACA 2014	40	KX373949	KX373921
	R	F14PA040	PACA 2014	40	KX373940	KX373922
	R	F14PA043	PACA 2014	8	KX373938	KX373923
	R	F14PA047	PACA 2014	40	KX373937	KX373924
	R	F14PA050	PACA 2014	40	KX373950	KX373925
	R	F14PA052	PACA 2014	40	KX373951	KX373926
	R	A01	PACA 2014	40	KX373941	KX373902
	Unknown	A03	PACA 2014	40	-	KX373903
	R	A04	PACA 2014	40	KX373942	KX373904
	Unknown	A07	PACA 2014	40	-	KX373905
	Unknown	F14PA145	PACA 2014	5	KX373947	-
	Unknown	F14PA149	PACA 2014	5	-	KX373918
RecVT-FR3	R	F15SA228	Sarthe 2015	10	KX373954	NT
RecVT-FR4	R	F14SA064	Sarthe 2014	10	KX373952	KX373935
RecVT-FR2	R	F14SA066	Sarthe 2014	10	KX373953	KX373936
	R	F14PR097	Paris 2014	20	KX373943	KX373927
	R	F14PR099	Paris 2014	20	KX373944	KX373928
	Unknown	F14PR100	Paris 2014	20	-	+
DWV-Fr3	P	F14PR102	Paris 2014	20	KX373956	KX373929
	Unknown	F14PR108	Paris 2014	20	+	KX373930
	Unknown	F14PR110	Paris 2014	20	-	KX373931
	Unknown	IT13AR029	Italy 2013	1	-	KX373908

	Unknown	IT13AR030	Italy 2013	1	-	KX373909
	Unknown	IT14AR135	Italy 2014	9	-	KX373932
	Unknown	IT14AR136	Italy 2014	4	-	KX373933
	Unknown	IT14AR137	Italy 2014	7	-	KX373934
	Unknown	AR14LP112	Argentina 2014	14	-	KX373917
	Unknown	AR14BB125	Argentina 2014	6	-	KX373910
	Unknown	AR14BB126	Argentina 2014	20	-	KX373911
	Unknown	AR14BB128	Argentina 2014	20	-	KX373912
	Unknown	AR14BB132	Argentina 2014	20	-	KX373913
	Unknown	CA15AL158	Canada	5	-	KX373901

Supplementary Table S2 - Primers used for sequencing. Numbers at the end are indicative for primer position in the genome from this study

Primer name	Primer sequence	Forward (F) or Reverse (R)	To use with	In
**DWV-UTR1f**	CGATTTATGCCTT(C/G)CATAGCG	F	DWV-1795r	This study
**DWV-ITR3f_1136**	TGGCCTTTAGTTGCGGAACC	F	DWV-Lp3r_1663	This study
**DWV-LP5f_1196**	AAAATGGCCTTTAGTTGCGG	F	DWV-CP5r_2689	This study
**DWV-Lp5r_1263**	TA(T/C)GAGTTAGAGTGTGT(C/T)AC	F	DWV-CP5r_2922	This study
**DWV-Lp3r_1663**	GGTACTAGGATCACGTTGTG	R	DWV-ITR3f_1136	This study
**DWV-1795r**	TACGTTCTTGCTCCAGCGCC	R	DWV- UTR1f	This study
**DWV-CP5r_2922**	GCATAAACATACTAGATACG	R	DWV-Lp5r_1263	This study
**DWV-CP5f_2649**	TGCATAGTTTAGCTTTAGGC	F	DWV-CP3r_4144	This study
**DWV-CP5r_2689**	ACCCGTATGAAATTGG	R	DWV-LP5f_1196	This study
**DWV-CP3f_4015**	CATTGTATGTTTACCTTCCC	F	DWV-Hel5r_5148	This study
**DWV-CP3f_4021**	TACGGATAAGGATATTGATC	F	DWV-Hel5r_6081	This study
**DWV-CP3f_4041**	CATTG(T/C)ATGTTTACCTTCCC	F	DWV-Hel5r_6081	This study
**DWV-CP3r_4144**	TACCCTGACGCTATCAATGG		DWV-CP5f_2649	This study
**DWV-CP3f_4199**	TATTGTTATCCGTTACTACG	F	DWV-Hel5r_6115	This study
**DWV-Hel5r_5148**	TACCTGATTACTACCTCTAC	R	DWV-CP3f_4015	This study
**DWV-5992f**	TCCTATTGCTGAATGTAGTC	F	DWV-6454r	[1]
**DWV-Hel5r_6056**	TTCAGACCATGTGGTCTCGG	R	DWV-CP3f_4021 or DWV-CP3f_4041	This study
**DWV-Hel5r_6081**	TTCAAGAAATTCATTATACGTC	R	DWV-CP3f_4021 or DWV-CP3f_4041	This study
**DWV-Hel5r_6115**	CATATACACAGGAGTTATCC	R	DWV-CP3f_4199	This study
**DWV-6285f**	GAGCGTACACTATGGTCAGA	F	DWV-6693r	[1]
**DWV-6454r**	CGAACTCATAACCTCATAAG	R	DWV-5992f	[1]

**DWV-6693r**	GTTCACGACGCTTACTACAC	R	DWV-6285f	[1]
**DWV-Hel3f_6558**	AGATGAAGAACTTATGGGCG	F	DWV-RdRp5r_8908	This study
**DWV-Hel3f_7326**	GCTGATCATTATGTGAATAGGC	F	DWV-Rd5r_7954 or DWV-Rd5r_8863	This study
**DWV-Rd5r_7954**	CCAATAGCTGAgTATGGtCGCC	R	DWV-Hel3f_7326	This study
**DWV-Rd5r_8863**	CTAAATATTCTAGTCTTACC	R	DWV-Hel3f_7326	This study
**DWV-Rd5f_8747**	TTCAGTTATCAACGACACAG		DWV-Rd3r_9134	This study
**DWV-Rd5r_8908**	CTAAGTAATACTGTCGAAA(T/C)GG	R	DWV-Hel3f_6558 or DWV-Hel3f_7326	This study
**MDF**	ATATTCACGGATTGTTTGAAAGA	F	MDR	[2]
**MDR**	CRCTAACATTCATGATAAGATCGTC	R	MDF	[2]
**DWV-Rd3r_9134**	CAGGTTAGCTAGAAACACAG	R	DWV-Rd5f_8747	This study
**DWV-RdRp3f_9316**	ATTAGGTTAGCTTGGTTAGG	F	Oligo dTs	This study
**DWV-RdRp3f_9324**	AGCTTGGTTAGGTATTACTG	F	Oligo dTs	This study
**DWV-ITR5r_318**	GCGGTCACATGGCAATCTAC	R	For 5' RACE	This study
**DWV-ITR5r_460**	TACTCGATACTGCAGTGGTC	R	For RT 5' RACE	This study

Supplementary Table S2 references

1 Berenyi, O. *et al.* Phylogenetic analysis of deformed wing virus genotypes from diverse geographic origins indicates recent global distribution of the virus. *Applied and Environmental Microbiology* **73**, 3605-3611, doi:10.1128/aem.00696-07 (2007).

2 Meeus, I., Smagghe, G., Siede, R., Jans, K. & de Graaf, D. C. Multiplex RT-PCR with broad-range primers and an exogenous internal amplification control for the detection of honeybee viruses in bumblebees. *J Invertebr Pathol* **105**, 200-203, doi:10.1016/j.jip.2010.06.012 (2010).

Supplementary Figure S1 - Neighbor-Joining tree of the complete genome sequences of DWV, VDV-1 and KV. This analysis involved 14 nucleotide sequences, 12 of which were obtained from the GenBank database and 2 that were obtained during this study (◆). The DWV and KV reference sequences are indicated by a red circle (●), VDV-1 are indicated by a yellow circle (●), and recombinant sequences are indicated by an orange circle (●). Evolutionary distances were computed using the Maximum Composite Likelihood method. The percentages of replicate trees in which the associated taxa clustered together in the bootstrap test (1000 replicates) are shown next to the branches[3]. All positions with less than 95% site coverage were eliminated. There were 9,858 positions in the final dataset.

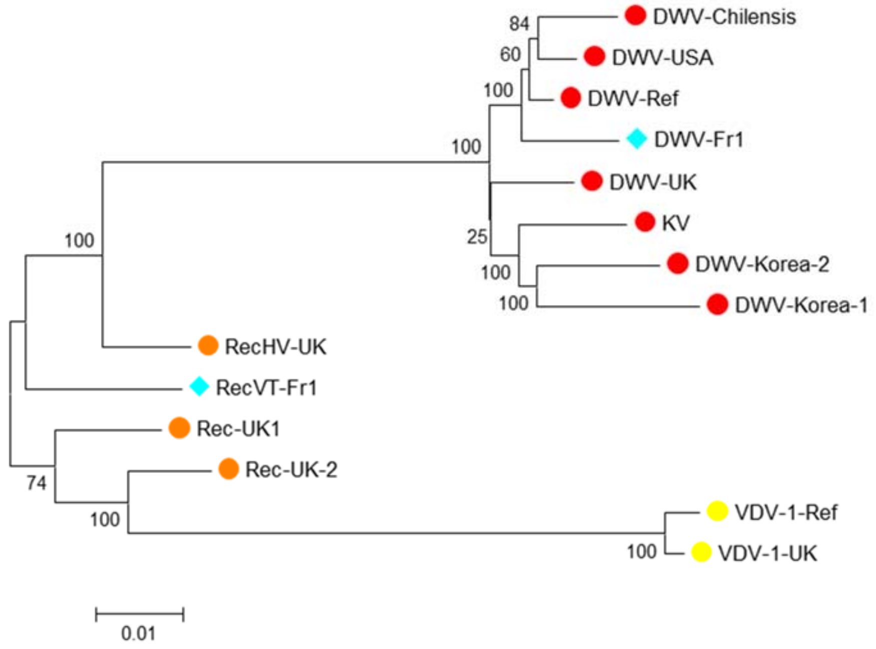

Supplementary Figure S2 - Distance probability a) to the nearest neighboring breakpoint, and b) of any site in the genome to the nearest breakpoint. The long dotted lines indicate the 5% significance threshold, while the short dotted lines indicate the 2.5% threshold. The continuous lines were obtained using our dataset.

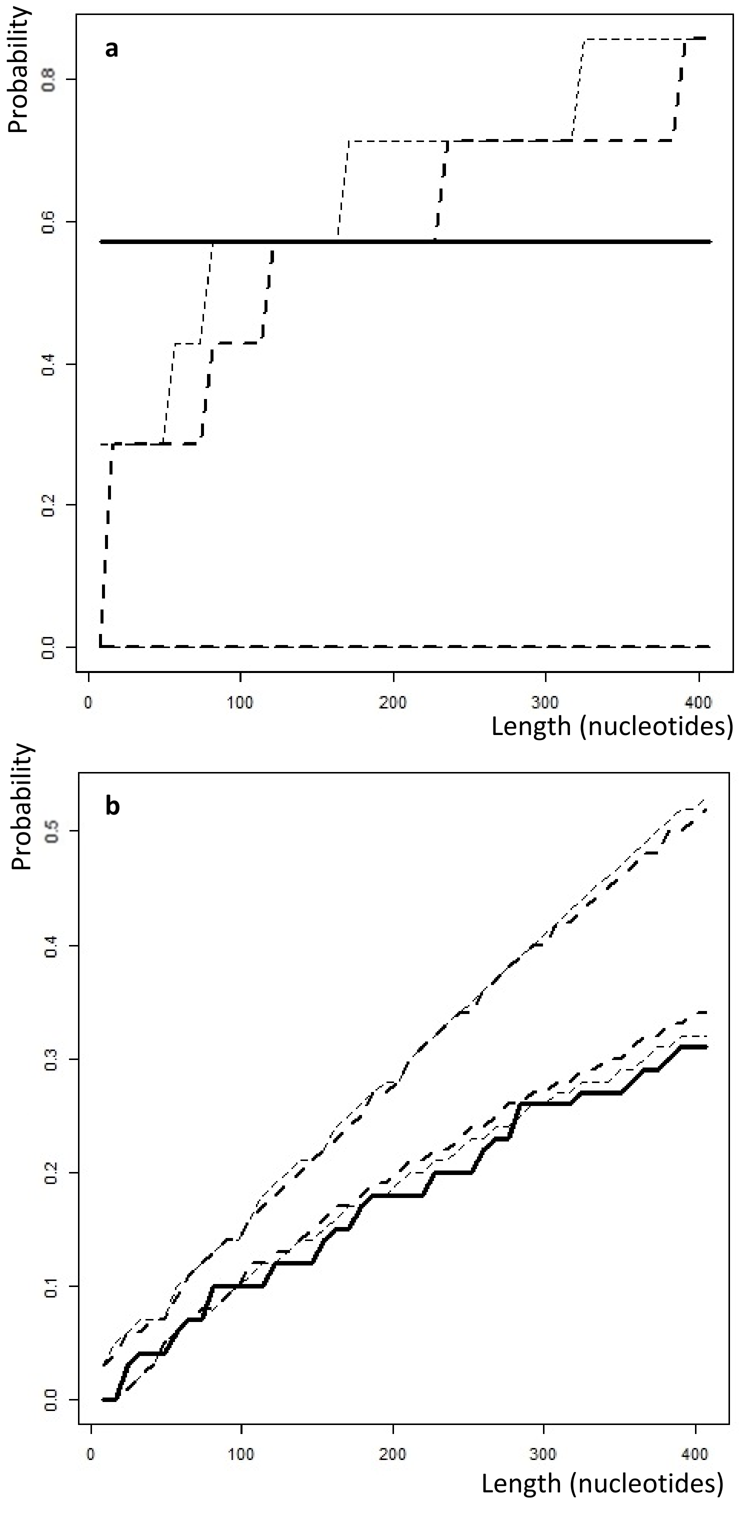

Supplement: Supplementary Material [file srep41045-s1.pdf]
